# Association Between Longitudinal Changes in Cardio-Ankle Vascular Index and Aortic Stenosis Progression in Dialysis Patients: A Retrospective Study

**DOI:** 10.7759/cureus.93263

**Published:** 2025-09-26

**Authors:** Shunsuke Todani, Kazuhiro Shimizu, Shuji Sato, Kohji Shirai, Atsuhito Saiki

**Affiliations:** 1 Division of Diabetes, Metabolism, and Endocrinology, Department of Internal Medicine, Toho University Graduate School of Medicine, Tokyo, JPN; 2 Division of Cardiology, Department of Internal Medicine, Toho University Sakura Medical Center, Sakura, JPN; 3 Department of Internal Medicine, Mihama Hospital, Chiba, JPN

**Keywords:** aortic stenosis, aortic valve replacement, arterial stiffness, cardio-ankle vascular index, hemodialysis

## Abstract

Objective

In dialysis patients, echocardiography often underestimates the progression of aortic stenosis (AS) due to blood pressure fluctuations and extensive calcification that obscure standard indices. We evaluated whether serial changes in the cardio-ankle vascular index (CAVI), a blood pressure-independent marker of arterial stiffness, can anticipate severe AS and guide the timing of valve replacement.

Methods

This single-center, retrospective study screened 1,169 maintenance dialysis patients (2015-2023). Forty-one patients who underwent surgical or transcatheter aortic valve replacement (AVR/TAVR) had valid CAVI measurements at four time points: 2 years and 1 year before surgery, immediately before surgery, and 1 year after surgery. The -2-year time point served as the baseline. Hemodynamic, echocardiographic, and laboratory data were analyzed using the Friedman and Wilcoxon tests. Severe AS progression was defined as a new mean pressure gradient (meanPG) ≥ 40 mmHg. Determinants of progression were identified using multivariable logistic regression.

Results

Median CAVI declined from 10.2 to 9.4 over the two years preceding surgery (p < 0.001) and rose to 11.1 one year post-AVR/TAVR (p < 0.001). Simultaneously, ejection time increased, ejection fraction decreased, and Vmax, maxPG, stroke volume, and stroke volume index (SVi) all rose significantly (p < 0.01 for each), with no significant changes in blood pressure or heart rate. Patients who progressed to severe AS showed a greater CAVI decline (ΔCAVI -2.90 vs. -0.67; p = 0.022) and higher Vmax (4.0 vs. 3.0 m/s; p < 0.001). ΔCAVI was the only independent predictor of severe AS progression (OR 0.51 per 1-unit decrease; 95% CI: 0.20-0.89; p = 0.013). Age and sex were not significant. CAVI and peak PG showed a modest inverse correlation (ρ = -0.35).

Conclusion

In dialysis patients, a marked preoperative decline in CAVI nearly doubles the risk of emergent severe AS and normalizes after valve replacement, indicating true hemodynamic responsiveness. Serial CAVI monitoring, simple, noninvasive, and reproducible, may offer an early warning signal and help optimize intervention timing when echocardiographic findings are ambiguous.

## Introduction

Aortic stenosis (AS) is increasingly common among the elderly and, if left untreated, can lead to worsening heart failure [1, 2], reduced survival [3-5], and sudden cardiac death [6-8]. In patients undergoing dialysis, chronic inflammation and disturbances in mineral metabolism, particularly involving calcium, phosphorus, and parathyroid hormone (PTH), accelerate vascular and valvular calcification, resulting in a higher prevalence of AS than in the general population [9-13]. Previous studies have reported that the prevalence of AS is approximately 3-5% among individuals aged ≥75 years in the general population, whereas dialysis cohorts demonstrate substantially higher rates, with estimates of ~7-15% for any AS and ~3-6% for moderate-to-severe disease [10, 11].

In patients undergoing dialysis, the reliability of echocardiographic indices is reduced due to heavy valvular calcification and substantial hemodynamic variability from intradialytic fluid shifts, which can obscure accurate assessment of AS severity [11].

Furthermore, some patients qualify for valve intervention despite only mild echocardiographic findings. These factors complicate the accurate evaluation of AS progression and pose challenges in determining optimal treatment strategies [14, 15]. Therefore, there is an urgent need for new, objective indicators that can assess AS progression earlier and more reliably.

The cardio-ankle vascular index (CAVI) is a noninvasive measure of arterial stiffness from the aortic valve to the tibial artery that is independent of blood pressure at the time of measurement [16]. CAVI has traditionally been used to assess atherosclerosis and cardiovascular risk. More recently, marked increases in CAVI following surgical or transcatheter aortic valve replacement (SAVR or TAVR) have been observed in patients with AS; this suggests that severe AS may lead to an underestimation of CAVI due to altered hemodynamics [17].

However, the longitudinal trajectory of CAVI during AS, especially in dialysis patients, remains poorly understood. If changes in CAVI reflect AS severity, the index could serve as a supplementary tool for identifying surgical indications in cases where echocardiographic evaluation is challenging. This study aimed to retrospectively investigate the relationship between longitudinal changes in CAVI and AS progression in dialysis patients, and to evaluate the potential of CAVI as a predictive marker for AS severity. Based on these considerations, we hypothesized that longitudinal changes in the cardio-ankle vascular index (ΔCAVI) precede the progression of AS in dialysis patients and may serve as a sensitive marker for identifying patients at risk of disease worsening and guiding timely intervention.

## Materials and methods

Participants

Between January 2015 and December 2023, a total of 1,169 patients undergoing maintenance dialysis at Seijinkai Mihama Hospital were screened. Among them, 69 patients who had undergone SAVR or TAVR for AS were identified.

Patients were excluded if CAVI measurements were missing at any of the four predefined time points (two years before surgery, one year before surgery, immediately before surgery, and one year after surgery) or if measurement errors were caused by conditions unrelated to AS (e.g., peripheral arterial disease). Specifically, patients with an extremely low ankle-brachial index (ABI < 0.9) or with undetectable pulse waves were considered to have PAD-related measurement errors and were excluded.

Ultimately, 41 patients with complete CAVI data at all four time points were included in this retrospective observational study. Baseline characteristics, including age at surgery, dialysis duration, height, dry weight, and BMI, were obtained from electronic medical records.

Echocardiography is routinely performed once per year, and CAVI is likewise measured annually as part of standard atherosclerosis screening in our dialysis cohort, rather than for research purposes. However, data obtained earlier than two years before surgery were too sparse and inconsistent across patients to allow reliable analysis. Consequently, the time point two years before surgery was designated as the baseline for longitudinal analysis. To ensure uniform comparability across patients, we prespecified four anchor time points relative to the date of valve replacement (-2 years, -1 year, immediately preoperative, and +1 year). When multiple measurements were available within a given interval, the value closest to the anchor time point was selected; interim values outside these windows were not analyzed to avoid imbalance in follow-up density.

**Figure 1:**
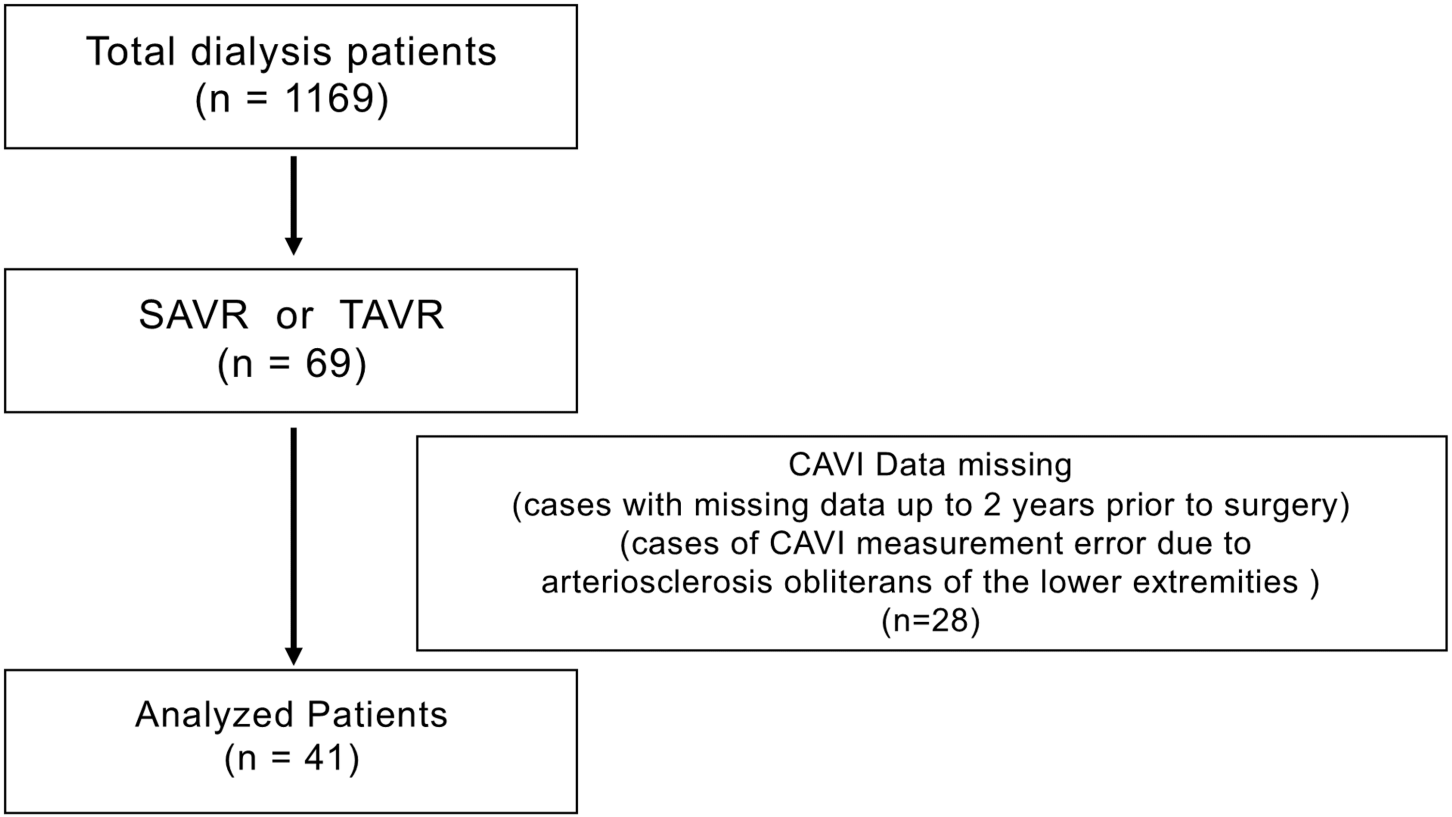
Patient selection flowchart. Of 1,169 maintenance dialysis patients, 69 underwent SAVR or TAVR. Of these, 28 were excluded due to missing CAVI data within 2 years prior to surgery or invalid measurements caused by lower-extremity arteriosclerosis obliterans, leaving 41 patients for analysis. SAVR: Surgical aortic valve replacement; TAVR: Transcatheter aortic valve replacement; CAVI: Cardio-ankle vascular index.

A flowchart of patient selection is shown in Figure 1.

CAVI and blood pressure measurement

Patients were examined in a quiet room maintained at a constant temperature. CAVI values were measured using a vascular screening system (VaSera1500; Fukuda Denshi Co., Ltd., Tokyo, Japan) according to a previously described method [16]. CAVI measurements were obtained by trained clinical laboratory technicians. Operators were not involved in AS severity evaluation or outcome assessment and were therefore blinded. Measurement procedures were standardized across all cases.

The CAVI was calculated using the following formula derived from the Bramwell-Hill equation:

\[ \text{CAVI} = a \left( \frac{2\rho}{\Delta P} \times \ln \left( \frac{P_s}{P_d} \right) \cdot \text{PWV}^2 \right) + b \]

where Ps and Pd represent systolic and diastolic blood pressures, respectively; ΔP is the pulse pressure (Ps - Pd); ρ is blood density; PWV is the pulse wave velocity from the aortic origin to the tibial-femoral artery junction; and a and b are constants. CAVI values were automatically calculated by the VaSera system.

In this study, systolic and diastolic blood pressures were measured on the right upper arm (non-shunt side), and both blood pressure and heart rate were recorded simultaneously with CAVI measurements using the VaSera1500.

Echocardiography　

Transthoracic echocardiography was performed at four time points (two years before surgery, one year before surgery, immediately before surgery, and one year after surgery) using a GE Logiq P6 ultrasound system (GE Healthcare, Tokyo, Japan). AS severity was graded according to current ESC/EACTS and ACC/AHA guidelines [18, 19]. The following parameters were measured at each time point: ejection time (ET), ejection fraction (EF), peak aortic jet velocity (Vmax), maximum pressure gradient (maxPG), stroke volume (SV), and stroke volume index (SVi). Mean pressure gradient (meanPG) and aortic valve area (AVA) were assessed only at the immediate preoperative examination. All measurements were conducted in accordance with the recommendations of the American Society of Echocardiography (ASE) and performed by experienced sonographers; results were confirmed by board-certified cardiologists. MeanPG and AVA were analyzed only at the immediate preoperative stage because these parameters were not systematically obtained during routine annual echocardiography at the dialysis clinic. Instead, they were available only from preoperative evaluations performed at cardiac surgery centers, and data were included in the analysis when such records could be confirmed.

Laboratory measurements

Laboratory data at each time point were obtained from routine pre-dialysis blood samples collected on the third dialysis-free day closest to each CAVI measurement.

The analyzed parameters included N-terminal pro-B-type natriuretic peptide (NT-proBNP), serum calcium (Ca), phosphorus (P), intact parathyroid hormone (i-PTH), total cholesterol (T-Cho), triglycerides (TG), HDL cholesterol (HDL-C), LDL cholesterol (LDL-C), albumin (Alb), cholinesterase (ChE), hemoglobin (Hb), and hemoglobin A1c (HbA1c). HbA1c was assessed only in patients with a prior diagnosis of diabetes.

All laboratory values were retrospectively retrieved from electronic medical records.

Statistical analysis

For variables with complete data at all four time points, temporal changes were assessed using the Friedman test, with Bonferroni correction applied for post hoc comparisons. For CAVI, only patients with complete data at all four time points were included, eliminating missingness. For other variables, available data were analyzed, and no imputation was performed.

The Wilcoxon signed-rank test was used to compare paired values between two years before surgery and immediately before surgery. Comparisons between the AS progression group (patients who newly developed meanPG ≥ 40 mmHg) and the non-progression group were performed using the Mann-Whitney U test for continuous variables.

As a supplementary analysis, Spearman’s rank correlation coefficient (ρ) and coefficient of determination (R²) were calculated using CAVI and maxPG values from the same individuals across the four time points to evaluate the relationship between CAVI and AS progression.

Finally, multivariable logistic regression analysis was conducted with ΔCAVI as the primary explanatory variable, adjusting for age and sex as potential confounders.

All statistical analyses were performed using JMP® software (SAS Institute, Cary, NC, USA), with p-values < 0.05 considered statistically significant. Bonferroni-adjusted p-values for all pairwise Wilcoxon comparisons are reported in Appendix 1. Median differences with 95% CIs (Hodges-Lehmann) are provided in Appendices 2-3.

Ethical considerations

This study was approved by the Ethics Committee of Toho University Sakura Medical Center (approval number: S23061), with Seijinkai Medical Corporation as a collaborating institution. As a retrospective study using existing data collected during routine clinical care, all data were anonymized to protect personal information. Information about the study was disclosed on the institutional website, providing potential participants the opportunity to opt out. No patients requested exclusion after disclosure; therefore, the representativeness of the study sample was not affected.

## Results

Clinical characteristics　

The clinical characteristics of the 41 patients included in the analysis are summarized in Table 1. The cohort was predominantly elderly, with a median age of 76 years (IQR: 71-82), a median dialysis duration of 9 years (IQR: 5-18), and a median BMI of 20.8 kg/m² (IQR: 19.5-24.0). Most patients were male (58.5%). Diabetic nephropathy was the most common primary cause of end-stage kidney disease. TAVR was performed slightly more frequently than SAVR.

**Table 1:** Clinical characteristics of the study population. Data are presented as median (IQR) or n (%); n = 41 for all variables. ESRD: End-stage renal disease; SAVR: Surgical aortic valve replacement; TAVR: Transcatheter aortic valve replacement.

Variable	Value
Demographics (n = 41)	
Age, years	76 (71-82)
Dialysis vintage, years	9 (5-18)
Male sex, n (%)	24 (58.5%)
Anthropometrics, median (IQR)	
Height, cm	161.0 (154.5-164.1)
Body weight, kg	54.0 (49.0-61.0)
BMI, kg/m²	20.8 (19.5-24.0)
Etiology of ESRD, n (%)	
Diabetic nephropathy	19 (46.3%)
Chronic glomerulonephritis	9 (22.0%)
Nephrosclerosis	6 (14.6%)
Polycystic kidney disease	3 (7.3%)
Others	2 (4.9%)
Unknown	2 (4.9%)
Operative method, n (%)	
SAVR	18 (43.9%)
TAVR	23 (56.1%)

Correlation analysis between CAVI and maxPG

**Figure 2:**
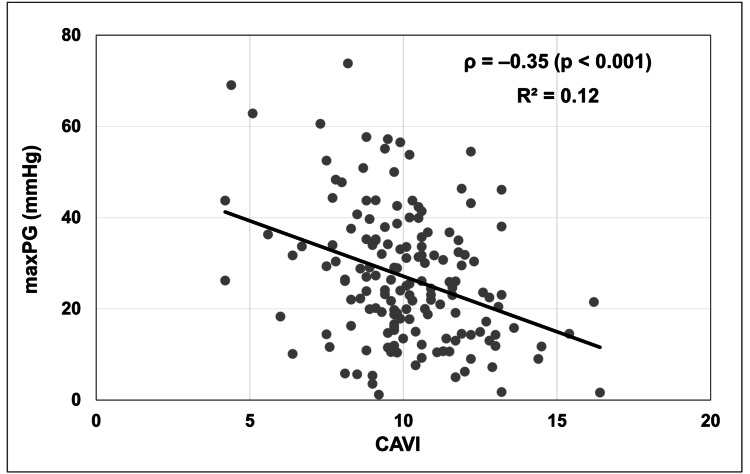
Correlation between CAVI and maxPG. Scatter plot of CAVI versus maxPG. A modest negative correlation was observed (Spearman’s ρ = -0.35, p < 0.001). A simple linear fit is overlaid to illustrate the trend (R² = 0.12). A total of 156 data points from 41 patients are shown, reflecting missing measurements at some time points. CAVI: Cardio-ankle vascular index; maxPG: Maximum pressure gradient.

As shown in Figure 2, CAVI and maxPG demonstrated a weak negative correlation across the four time points (Spearman’s ρ = -0.35, p < 0.001; R² = 0.12). Because the analysis involved repeated measurements from the same individuals (i.e., non-independent data), the R² value was calculated for reference only and should be interpreted with caution. Nonetheless, the trend of lower CAVI values corresponding to higher maxPG visually supports the main finding regarding the association between ΔCAVI and AS severity.

Changes in clinical and cardiac parameters between two years preoperatively and immediately before surgery

As shown in Table 2, comparison of key clinical and echocardiographic parameters between two years preoperatively and immediately before surgery using the Wilcoxon signed-rank test revealed significant changes in several cardiac and hemodynamic indicators. In addition to p-values, median changes with 95% confidence intervals (Hodges-Lehmann) for the main outcomes (CAVI, ET, EF, and maxPG) are reported here; the full set for all parameters is summarized in Appendix 2.

**Table 2:** Changes in CAVI, echocardiographic, and laboratory parameters from two years before AVR to immediately before AVR. Changes in CAVI, echocardiographic, and laboratory parameters from two years before AVR in 41 maintenance dialysis patients. Data are presented as median (IQR). P-values were calculated using two-sided Wilcoxon signed-rank tests for paired comparisons; p < 0.05 was considered statistically significant, and an asterisk (*) indicates a statistically significant p-value. Missing values are indicated by “-”. AVR: Aortic valve replacement; CAVI: Cardio-ankle vascular index; ET: Ejection time; EF: Ejection fraction; V: Peak aortic jet velocity; maxPG: Maximum pressure gradient; SV: Stroke volume; SVi: Stroke volume index; sBP: Systolic blood pressure; dBP: Diastolic blood pressure; meanBP: Mean blood pressure; HR: Heart rate; AVA: Aortic valve area; meanPG: Mean pressure gradient; NT-proBNP: N-terminal pro-B-type natriuretic peptide; Ca: Calcium; P: Phosphorus; i-PTH: Intact parathyroid hormone; T-Cho: Total cholesterol; TG: Triglycerides; HDL-C: High-density lipoprotein cholesterol; LDL-C: Low-density lipoprotein cholesterol; Alb: Albumin; ChE: Cholinesterase; Hb: Hemoglobin; HbA1c: Glycated hemoglobin.

	2 years before AVR	Immediately before AVR (Baseline)	p-value
BMI (kg/m²)	-	20.8 (19.5-24.0)	-
CAVI	10.2 (9.1-11.7)	9.4 (7.8-10.5)	<0.001*
ET (ms)	297 (279-321)	316 (285-339)	0.003*
EF (%)	63.1 (58.6-66.3)	59.0 (53.5-63.0)	0.002*
V (m/s)	2.2 (1.8-2.6)	4.0 (3.0-4.2)	<0.001*
maxPG (mmHg)	20.1 (12.5-27.2)	36.8 (31.7-43.8)	<0.001*
SV (mL)	56.5 (45.5-65.9)	70.0 (57.0-77.9)	0.002*
SVi (mL/m²)	34.4 (29.2-41.5)	44.4 (39.3-50.4)	0.003*
sBP (mmHg)	144 (124-159)	143 (135-159)	0.46
dBP (mmHg)	80 (72-91)	78 (69-89)	0.151
meanBP (mmHg)	109 (100-124)	109 (102-122)	0.591
HR (bpm)	65 (59-75)	65 (59-75)	0.567
AVA (cm²)	-	0.8 (0.7-0.9)	-
meanPG (mmHg)	-	37.5 (31.8-41.8)	-
NT-proBNP (pg/mL)	15,400 (4,710-25,650)	22,145 (11,789-33,933)	0.094
Ca (mg/dL)	8.4 (8.1-8.8)	8.7 (8.3-9.1)	0.018*
P (mg/dL)	5.8 (4.6-6.9)	5.1 (4.6-6.1)	0.017*
i-PTH (pg/mL)	211 (123-241)	153 (105-176)	0.022*
T-Cho (mg/dL)	155 (140-184)	152 (133-188)	0.619
TG (mg/dL)	95 (56-125)	83 (52-106)	0.101
HDL-C (mg/dL)	50 (38-63)	51 (40-67)	0.35
LDL-C (mg/dL)	74 (62-99)	73 (60-94)	0.538
Alb (g/dL)	3.5 (3.4-3.6)	3.4 (3.3-3.7)	0.302
ChE (U/L)	216 (198-266)	228 (187-259)	0.135
Hb (g/dL)	11.4 (10.5-11.8)	11.0 (10.1-11.3)	0.018*
HbA1c (%)	5.7 (5.3-6.2)	6.0 (5.5-6.4)	0.754

Specifically, CAVI significantly decreased from 10.2 (9.1-11.7) to 9.4 (7.8-10.5) (p < 0.001). Ejection time (ET) increased from 296.5 (279.0-320.8) ms to 316.0 (285.0-338.5) ms (p = 0.0030), and ejection fraction (EF) declined from 63.1% (58.6-66.3) to 59.0% (53.5-63.0) (p = 0.0015). Additionally, Vmax, maximum pressure gradient (maxPG), stroke volume (SV), and stroke volume index (SVi) all showed statistically significant increases (p < 0.01 for all).

No significant changes were observed in systolic, diastolic, or mean blood pressure, or in heart rate.

Regarding biochemical markers, significant changes were found in serum calcium (Ca), phosphorus (P), intact parathyroid hormone (i-PTH), and hemoglobin (Hb), all with p < 0.05. However, NT-proBNP, triglycerides (TG), total cholesterol (T-Cho), HDL-C, LDL-C, albumin (Alb), cholinesterase (ChE), and HbA1c showed no significant differences.

Longitudinal analysis　

Using the Friedman test, significant differences across the four time points were observed in 10 parameters: CAVI, ET, EF, Vmax, SV, SVi, NT-proBNP, Ca, TG, and ChE (all p < 0.05).

Among these, CAVI showed a significant decline from a median of 10.2 (9.1-11.7) two years before surgery to 9.4 (7.8-10.5) immediately before surgery (p < 0.001). A significant decrease was also observed between two years and one year before surgery (median 9.3 (8.3-10.0), p < 0.001). However, there was no significant difference between one year before surgery and immediately before surgery (9.3 → 9.4, p = 0.503). In contrast, CAVI significantly increased to 11.1 (10.4-12.2) one year after surgery compared to the immediate preoperative value (p < 0.001).

**Figure 3:**
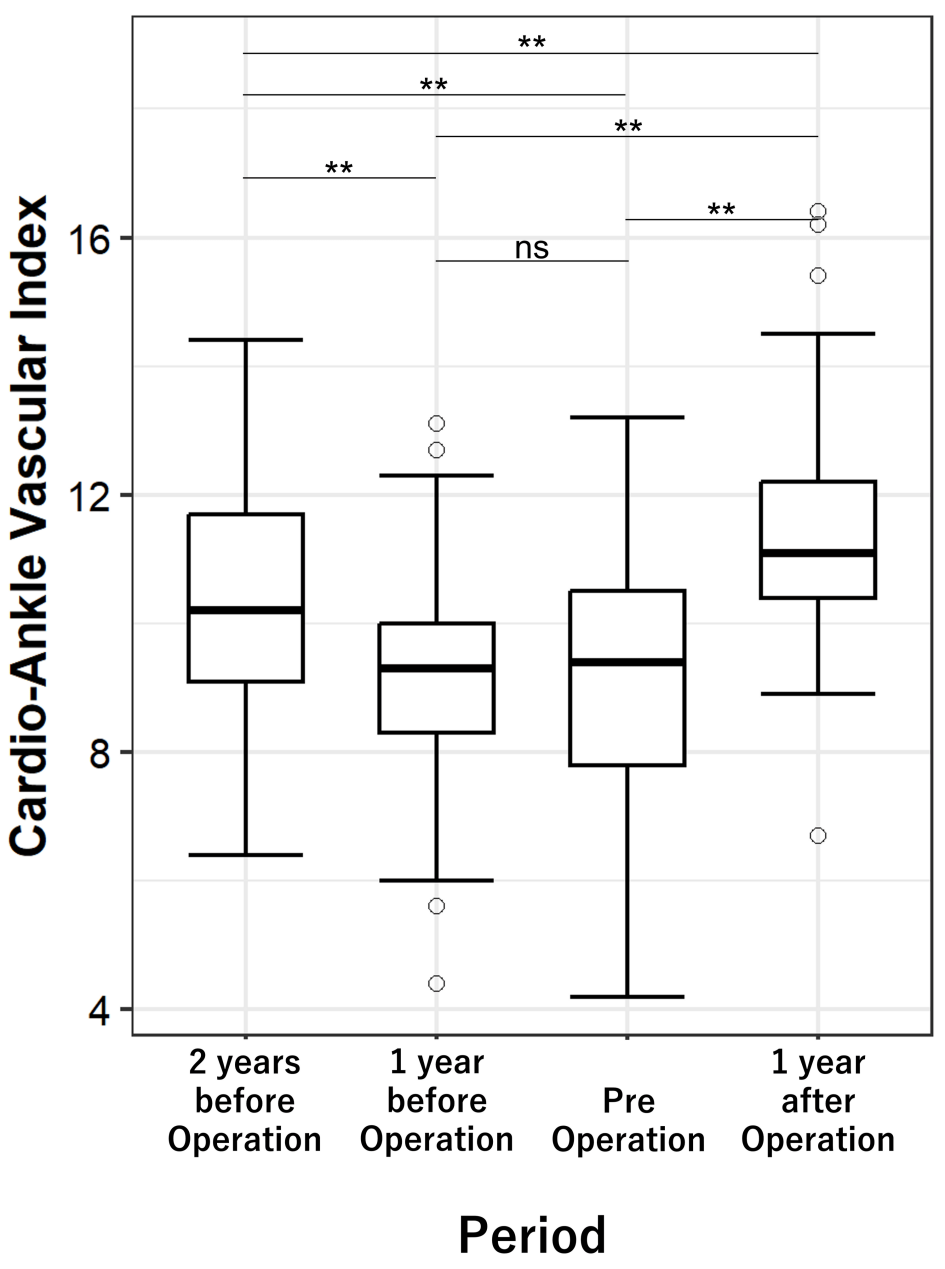
Changes in CAVI from 2 years pre- to 1 year post-operation. Box plots display the median and IQR; whiskers extend to 1.5×IQR, and circles indicate outliers. Overall differences across the four time points were assessed using the Friedman test, and pairwise post hoc comparisons were performed with Wilcoxon signed-rank tests and Bonferroni correction. ** indicates Bonferroni-corrected p < 0.01; ns = not significant. Data are from 41 patients. CAVI: Cardio-ankle vascular index.

Figure 3 presents a box-and-whisker plot of CAVI at the four time points. The box indicates the IQR, with the horizontal line representing the median. Whiskers denote the range within 1.5×IQR, and outliers are shown as open circles. Overall significance was assessed using the Friedman test (p < 0.001), and Bonferroni correction was applied for post hoc comparisons. Bonferroni-adjusted p-values for all pairwise time-point comparisons are shown in Appendix 1.

Comparison of patient background based on new onset of meanPG ≥ 40 mmHg

Table 3 summarizes the baseline characteristics of patients who newly developed a mean pressure gradient (meanPG) ≥ 40 mmHg between two years before surgery and immediately before surgery (AS progression group) versus those who did not (non-progression group). The Mann-Whitney U test was applied for continuous variables, and Fisher’s exact test for binary variables, with statistical significance defined as p < 0.05. The between-group median difference in ΔCAVI with its 95% confidence interval, together with effect sizes for other key variables, is presented in Appendix 3.

**Table 3:** Comparison of variables between patients with and without severe AS progression (mean PG ≥ 40 mmHg). Data are presented as median (IQR) or number (%). Between-group comparisons were performed using the two-sided Mann-Whitney U test; p < 0.05 was considered statistically significant, and an asterisk (*) indicates statistical significance. The progression group included 9 patients, and the non-progression group included 19 patients. AS: Aortic stenosis; PG: Pressure gradient; ΔCAVI: Change in cardio-ankle vascular index; CAVI: Cardio-ankle vascular index; V: Peak aortic jet velocity; EF: Ejection fraction.

	PG < 40 mmHg	PG ≥ 40 mmHg	p-value
ΔCAVI	-0.7 (-1.6 - -0.1)	-2.9 (-3.8 - -1.1)	0.022*
V (m/s)	3.0 (2.8-3.3)	4.0 (4.0-4.0)	<0.001*
EF (%)	58.5 (54.7-64.7)	54.0 (47.0-56.0)	0.055
ET (ms)	317 (281-343)	336 (292-359)	0.245
SVi (mL/m²)	45.0 (40.0-52.0)	44.4 (35.3-50.0)	0.588
NT-proBNP (pg/mL)	27,200 (8,620-34,450)	26,203 (14,000-31,366)	0.821

ΔCAVI was significantly greater in the AS progression group, with a median (IQR) of -2.90 (-3.80 to -1.10), compared to -0.67 (-1.55 to -0.05) in the non-progression group (p = 0.0221). Additionally, Vmax was significantly higher in the AS progression group (4.0 (4.0-4.0) m/s) than in the non-progression group (2.97 (2.73-3.28) m/s, p < 0.001). No significant differences were found in other factors, including age and sex.

Multivariable logistic regression analysis

A multivariable logistic regression analysis was conducted to identify predictors of AS progression, defined as the new onset of meanPG ≥ 40 mmHg between two years before surgery and immediately prior to surgery. ΔCAVI was included as the primary explanatory variable, while age and sex were entered as potential confounders (Table 4).

**Table 4:** Multivariate logistic regression identifying predictors of severe AS progression. n = 28; model R² = 0.2977; model p = 0.0150. Binary logistic regression was performed with mean PG ≥ 40 mmHg as the dependent variable. A significance level of α = 0.05 was applied. An asterisk (*) indicates a statistically significant p-value. AS: Aortic stenosis; PG: Pressure gradient; ΔCAVI: Change in cardio-ankle vascular index.

Variable	OR	95% CI	p-value
Men	0.255	0.03-1.91	0.181
Age	1.054	0.92-1.27	0.468
ΔCAVI	0.508	0.20-0.89	0.013*

Although Vmax also differed significantly between the progression and non-progression groups (p < 0.001), it was excluded from the final model due to strong multicollinearity with meanPG (Spearman’s ρ = 0.89, p < 0.0001).

The analysis identified ΔCAVI as the only independent predictor of AS progression, with an OR of 0.508 (95% CI: 0.198-0.886, p = 0.0128). This indicates that each one-unit decrease in ΔCAVI nearly doubles the risk of AS progression (1 / 0.508 ≈ 1.97). Neither age (OR: 1.054, 95% CI: 0.923-1.270, p = 0.468) nor sex (OR: 0.255, 95% CI: 0.028-1.940, p = 0.181) was significantly associated with AS progression.

## Discussion

In this study, we conducted a detailed longitudinal analysis of the CAVI in maintenance hemodialysis patients and evaluated whether changes in CAVI (ΔCAVI) reflect the progression of AS. Our findings demonstrated that CAVI values declined progressively from two years prior to surgery until just before the procedure, preceding the clinical manifestation of severe AS, and significantly increased after surgery. These results suggest that CAVI, a noninvasive and rapid test requiring no specialized skills, may serve as a sensitive marker for detecting early hemodynamic changes associated with AS and may rapidly normalize after valve replacement.

As a supplementary analysis, we examined the Spearman rank correlation between CAVI and peak pressure gradient (maxPG) measured at four time points in the same patients. Although a weak negative correlation was observed (Spearman’s ρ = -0.35, p < 0.001), the strength of the association was limited. Nevertheless, this trend visually supported the primary analysis using ΔCAVI, suggesting that reductions in CAVI may precede AS progression, as reflected by increases in meanPG. These findings provide complementary evidence for our hypothesis that CAVI decline serves as an early marker of AS progression. However, because the correlation was modest, future studies should use statistical methods that better handle repeated measurements to clarify this relationship.

Typically, CAVI increases with age and the progression of arteriosclerosis [20]. It is widely used as a marker of arterial stiffness and reflects various cardiovascular risk factors, including diabetes, hypertension, dyslipidemia, and sleep apnea. Furthermore, appropriate therapeutic interventions have been shown to lower CAVI, underscoring its usefulness in monitoring vascular damage and evaluating treatment effectiveness [21]. Several prospective studies and meta-analyses have proposed a CAVI cutoff of ≥9.0 for predicting the onset of cardiovascular disease. This prognostic value has been corroborated by international research, and elevated CAVI has also been linked to worse outcomes in dialysis patients [22, 23].

In contrast, our study identified a “reversal phenomenon” in this high-risk population: despite the expectation of advanced arteriosclerosis, CAVI decreased as AS progressed and then increased after valve replacement. We interpret the preoperative decline in CAVI as a hemodynamic underestimation rather than a true regression of structural arterial stiffness. With progressive AS, forward flow decreases and ET is prolonged, which blunts and delays the early systolic upstroke of the peripheral arterial waveform; under these conditions, the device algorithm identifies the onset of the pulse later in time, effectively inflating the inferred transit time between recording sites. The reason for this phenomenon has been reported to be a reduction in the estimated pulse-wave propagation speed, which consequently decreases CAVI [17, 24, 25]. After valve replacement, restoration of forward flow shortens ET and steepens the upstroke, allowing earlier detection of pulse onset and unmasking the higher underlying arterial stiffness as a postoperative rise in CAVI [17]. In line with these reports, our findings demonstrated significant changes in both CAVI and ET as AS advanced, indicating that AS directly affects hemodynamic conditions captured by CAVI. Moreover, in dialysis patients, advanced vascular stiffness and calcification may limit further decreases in CAVI, leading to a plateau as values approach a certain threshold. This vascular background could partly explain the stabilization observed between one year before and immediately prior to surgery. Previous reports demonstrating postoperative increases in CAVI after SAVR/TAVR were conducted primarily in general AS populations, with dialysis status seldom specified [17]. By focusing on maintenance dialysis patients, the present study extends prior work with dialysis-specific longitudinal data.

Furthermore, in the multivariable logistic regression analysis using ΔCAVI as a continuous variable, ΔCAVI was identified as an independent predictor of AS progression (defined as meanPG ≥ 40 mmHg), with nearly a two-fold increase in risk for each 1-unit decrease. Neither age nor sex showed significant associations, suggesting that CAVI may serve as a sensitive indicator of AS progression, independent of demographic characteristics.

A key finding of this study is that changes in CAVI occur early in the course of AS progression. Specifically, the median CAVI declined from 10.2 two years before surgery to 9.4 immediately prior to surgery, and then increased to 11.1 one year after surgery. Notably, traditional blood-based markers such as BNP, calcium, and phosphorus did not show significant associations with AS progression, suggesting that CAVI may serve as a more sensitive and earlier marker. Even in the absence of clinical symptoms or abnormalities in echocardiographic or laboratory findings, CAVI may capture early hemodynamic disturbances, potentially functioning as an early warning indicator for AS deterioration.

Echocardiographic assessments in dialysis patients often suffer from limited reproducibility, and sole reliance on meanPG or AVA may not provide an accurate evaluation of AS severity [11]. Furthermore, many dialysis facilities lack on-site cardiologists or cardiac surgeons, which can delay timely intervention.

Given that CAVI is noninvasive, quick to perform, and simple to administer, it may serve as a useful supplementary tool for monitoring AS progression in this high-risk population. A downward trend in serial CAVI measurements should raise suspicion for AS progression and prompt timely referral to a specialist.

In practice, ΔCAVI should complement rather than replace guideline-based triggers. A consistent downward trend on serial CAVI, especially in conjunction with symptoms or evidence of ET prolongation, may reasonably prompt earlier echocardiographic reassessment or specialist referral. The present study is underpowered to propose numeric thresholds; prospective validation is warranted.

Moreover, because CAVI is calculated automatically using a standardized algorithm, it offers high measurement reproducibility, as noted by Budoff MJ et al. [26]. Future large-scale prospective studies will be necessary to validate the clinical utility and predictive value of CAVI in managing AS among dialysis patients.

Because patients with ABI < 0.9 or non-detectable distal pulse waves were excluded to avoid PAD-related measurement error, our findings are most generalizable to patients in whom CAVI can be reliably acquired. In patients with PAD, damped distal waveforms, altered reflection sites, and segmental occlusive lesions may bias pulse-onset detection and the behavior of ΔCAVI; thus, performance and thresholds may differ. Future studies should include low-ABI cohorts with waveform-quality filters and PAD-specific sensitivity analyses to define appropriate use and interpretation of serial CAVI.

This study has several limitations. First, this retrospective, single-center study included only dialysis patients with AS who had complete CAVI measurements at four time points. The small sample size and limited inclusion criteria may have introduced selection bias and reduced statistical power, potentially limiting the ability to detect modest associations and affecting the generalizability of the findings. Therefore, the results should be interpreted with caution. Moreover, because this study was restricted to dialysis patients with AS, its findings may not be directly applicable to non-dialysis AS populations. Further studies in broader cohorts are warranted.

Second, data from more than two years prior to surgery were unavailable, making it difficult to assess long-term trends in CAVI. In most cases, annual echocardiographic follow-ups were performed as part of routine care, and reliable preoperative data beyond two years were rarely accessible.

Third, critical echocardiographic parameters such as meanPG and AVA were available only at the preoperative time point, which prevented the evaluation of their longitudinal changes.

Fourth, the study included only patients who underwent SAVR or TAVR, lacking a non-surgical comparison group. This limits the broader applicability of the findings. Stratified analyses by surgical modality (SAVR vs. TAVR) or baseline calcification severity were not feasible due to the limited sample size. These exploratory approaches warrant future investigation.

Fifth, residual confounding due to dialysis duration, mineral metabolism (calcium, phosphorus, i-PTH), and vascular calcification could not be fully excluded and represents a study limitation.

Finally, patients with measurement errors due to extremely low ankle-brachial index (ABI) or undetectable pulse waves, often resulting from peripheral arterial disease, were excluded. Therefore, caution is necessary when applying these results to patients with concomitant peripheral arterial disease.

To address these limitations, future prospective studies and multicenter collaborations are needed.

## Conclusions

In conclusion, this study demonstrated that a decrease in the CAVI is significantly associated with the progression of AS in patients undergoing dialysis. Serial CAVI monitoring may serve as a valuable supplementary marker for the early detection of AS progression and may support surgical decision-making, particularly when echocardiographic follow-up is challenging in this population. However, these results should be interpreted with caution given the retrospective, single-center design, the limited sample size, and the exclusion of patients with PAD-related measurement errors, which may restrict generalizability. Prospective, multicenter studies are warranted to validate these findings and to establish optimal clinical strategies for incorporating CAVI into AS management.

## Appendices

Appendix 1

**Table 5:** Bonferroni-adjusted p-values for pairwise Wilcoxon signed-rank tests following Friedman analysis across four time points. Bonferroni-adjusted p-values for multiple pairwise comparisons of CAVI, echocardiographic, and laboratory parameters across four time points (2 years pre-operation, 1 year pre-operation, immediately pre-operation, and 1 year post-operation) in 41 maintenance dialysis patients. An asterisk (*) indicates statistical significance at p < 0.05. AVR: Aortic valve replacement; CAVI: Cardio–ankle vascular index; ET: Ejection time; EF: Ejection fraction; V: Peak aortic jet velocity; maxPG: Maximum pressure gradient; SV: Stroke volume; SVi: Stroke volume index; sBP: Systolic blood pressure; dBP: Diastolic blood pressure; meanBP: Mean blood pressure; HR: Heart rate; NT-proBNP: N-terminal pro-B-type natriuretic peptide; Ca: Calcium; P: Phosphorus; i-PTH: Intact parathyroid hormone; T-Cho: Total cholesterol; TG: Triglycerides; HDL-C: High-density lipoprotein cholesterol; LDL-C: Low-density lipoprotein cholesterol; Alb: Albumin; ChE: Cholinesterase; Hb: Hemoglobin; HbA1c: Glycated hemoglobin.

	2 yrs pre-ope vs. 1 yr pre-ope	2 yrs pre-ope vs. Pre-ope	1 yr pre-ope vs. Pre-ope	2 yrs pre-ope vs. 1 yr post-ope	1 yr pre-ope vs. 1 yr post-ope	Pre-ope vs. 1 yr post-ope
CAVI	<0.001*	<0.001*	1	<0.001*	<0.001*	<0.001*
ET (ms)	0.046*	0.018*	1	1	0.547	0.048*
EF (%)	1	0.009*	0.049*	0.184	0.407	1
V (m/s)	0.039*	<0.001*	0.001*	1	0.034*	<0.001*
maxPG (mmHg)	0.017	<0.001*	0.001*	1	0.002*	<0.001*
SV (mL)	1	0.014*	0.007*	1	1	<0.001*
SVi (mL/m²)	1	0.018*	0.016*	1	1	0.002*
sBP (mmHg)	1	1	1	1	1	1
dBP (mmHg)	0.686	0.906	1	0.12	1	1
meanBP (mmHg)	1	1	1	1	1	1
HR (bpm)	1	1	1	1	1	1
NT-proBNP (pg/mL)	0.215	0.566	0.531	1	1	1
Ca (mg/dL)	1	0.106	0.667	1	0.494	0.038*
P (mg/dL)	1	0.1	0.372	1	1	0.035*
i-PTH (pg/mL)	1	0.133	0.404	1	1	1
T-Cho (mg/dL)	1	1	1	0.692	0.328	1
TG (mg/dL)	1	0.607	0.177	1	1	0.001*
HDL-C (mg/dL)	0.699	1	1	1	0.825	1
LDL-C (mg/dL)	1	1	1	1	1	1
Alb (g/dL)	1	1	1	0.495	0.547	1
ChE (U/L)	1	0.812	0.040*	0.412	0.006*	1
Hb (g/dL)	1	0.108	0.031*	1	1	0.479
HbA1c (%)	0.171	1	0.358	0.229	1	1

Appendix 2

**Table 6:** Median changes, 95% CIs, and p-values for all 23 clinical, echocardiographic, and laboratory parameters from 2 years before AVR to immediately before AVR Effect sizes (median changes with 95% confidence intervals) in CAVI, echocardiographic, and laboratory parameters from two years before AVR to immediately before AVR in 41 maintenance dialysis patients. Effect sizes were calculated using the Hodges–Lehmann estimator (HL). Data are presented with 95% confidence intervals (CI). P-values were calculated using two-sided Wilcoxon signed-rank tests for paired comparisons; p < 0.05 was considered statistically significant, and an asterisk (*) indicates a statistically significant p-value. Missing values were analyzed using available pairs only. AVR: Aortic valve replacement; CAVI: Cardio-ankle vascular index; ET: Ejection time; EF: Ejection fraction; V: Peak aortic jet velocity; maxPG: Maximum pressure gradient; SV: Stroke volume; SVi: Stroke volume index; sBP: Systolic blood pressure; dBP: Diastolic blood pressure; meanBP: Mean blood pressure; HR: Heart rate; NT-proBNP: N-terminal pro-B-type natriuretic peptide; Ca: Calcium; P: Phosphorus; i-PTH: Intact parathyroid hormone; T-Cho: Total cholesterol; TG: Triglycerides; HDL-C: High-density lipoprotein cholesterol; LDL-C: Low-density lipoprotein cholesterol; Alb: Albumin; ChE: Cholinesterase; Hb: Hemoglobin; HbA1c: Glycated hemoglobin.

	Median change (HL)	95% CI low	95% CI high	p-value	n (pairs)
CAVI	-1.03	-1.70	-0.55	<0.001*	41
ET (ms)	20	9	30	0.002*	34
EF (%)	-5.64	-9.35	-2.33	0.001*	39
V (m/s)	1.41	1.08	1.75	<0.001*	39
maxPG (mmHg)	17.47	12.88	22.74	<0.001*	39
Sv (mL)	13.37	3.50	23.19	0.009*	38
Svi (mL/m²)	9.33	2.94	15.72	0.004*	39
sBP (mmHg)	2.5	-4	10.5	0.464	41
dBP (mmHg)	-2.5	-6	1	0.153	41
meanBP (mmHg)	1	-4	6.5	0.595	41
HR (bpm)	-0.5	-2.5	2	0.571	41
NT-proBNP (pg/mL)	7067	-3199	15476	0.097	23
Ca (mg/dL)	0.3	0.05	0.55	0.018*	41
P (mg/dL)	-0.75	-1.35	-0.15	0.017*	41
i-PTH (pg/mL)	-59.6	-109	-8.5	0.024*	27
T-Cho (mg/dL)	-2	-11	7.5	0.624	41
TG (mg/dL)	-10.5	-24	1.5	0.102	41
HDL-C (mg/dL)	2	-2	5.5	0.321	41
LDL-C (mg/dL)	-1.5	-7	4	0.542	41
Alb (g/dL)	-0.05	-0.2	0.1	0.305	41
ChE (U/L)	-13.4	-25.5	3.5	0.137	41
Hb (g/dL)	-0.55	-1	-0.1	0.018*	41
HbA1c (%)	0.1	-0.3	0.65	0.660	17

Appendix 3

**Table 7:** Between-group differences in ΔCAVI and other key parameters (progression vs. non-progression groups). Between-group differences were analyzed using the Mann–Whitney U test. Effect sizes are expressed as median differences calculated using the Hodges–Lehmann estimator (HL), with 95% confidence intervals (CI). An asterisk (*) indicates statistical significance at p < 0.05. ΔCAVI: Change in cardio-ankle vascular index; V: Peak aortic jet velocity; EF: Ejection fraction; ET: Ejection time; SVi: Stroke volume index; NT-proBNP: N-terminal pro-B-type natriuretic peptide.

	HL (median diff)	95% CI low	95% CI high	p-value	n (Prog)	n (Non-prog)
ΔCAVI	-1.8	-3.4	-0.4	0.022*	9	19
V (m/s)	1.03	0.75	1.25	<0.001*	9	19
EF (%)	-8.8	-15.1	0	0.055	9	19
ET (ms)	18	-13	66	0.245	9	16
SVi (mL/m²)	-3.58	-14.05	5.19	0.588	9	19
NT-proBNP (pg/mL)	1575.83	-17,900.00	13,144.00	0.821	8	15
